# Phototoxic drug eruption induced by vandetanib used for the treatment of metastatic medullary thyroid cancer^☆^

**DOI:** 10.1016/j.abd.2021.08.010

**Published:** 2022-07-09

**Authors:** Ecem Bostan, Duygu Gulseren, Ozay Gokoz

**Affiliations:** a Department of Dermatology and Venereology, Hacettepe University, Faculty of Medicine, Ankara, Turkey; b Department of Pathology, Hacettepe University, Faculty of Medicine, Ankara, Turkey

Dear Editor,

Vandetanib is an oral tyrosine kinase inhibitor that specifically targets Rearranged During-Transfection (RET) proto-oncogene, Epidermal Growth Factor Receptor (EGFR), and Vascular Endothelial Grow Factor Receptor (VEGFR-2).^1^ Vandetanib is approved for the treatment of metastatic or locally advanced Medullary Thyroid Carcinoma (MTC).^1^ Herein, we would like to report a case of phototoxic drug eruption induced by vandetanib used for metastatic MTC.

A 54-year-old man with a history of MTC, presented with an erythematous, well-defined cutaneous eruption involving the face, neck, and hands. The cutaneous eruption had started from the dorsal parts of both hands with a burning, prickling, and itching sensation one month ago. Within a week, the erythematous cutaneous eruption spread to the face and neck with no involvement of the sun-protected areas. He was diagnosed with MTC 5-years ago. Subsequently, he had bilateral total thyroidectomy along with central and right neck dissection. Positron Emission Tomography/Computed Tomography (PET/CT) revealed findings consistent with lytic bone metastases. He was then treated with 2 cycles of radioactive lutetium Lu-177 dotatate therapy along with zoledronic acid monthly for seven months. Since he showed partial response to radioactive therapy, oral vandetanib 300 mg per day was started for metastatic MTC treatment. No other recently administered systemic or topical photosensitizer was noted. Two months after the start of oral vandetanib, he developed erythematous, photodistributed papules and plaques involving both dorsal hands/feet, neck, and face (Fig. 1, with the permission of the patient). Pre-diagnoses of phototoxic or photoallergic drug eruption associated with vandetanib were considered. A 4 mm punch biopsy was taken from the dorsal hand. Prominent vacuolar degeneration and hyperpigmentation were observed in the epidermis; extravasated erythrocytes, coat-sleeve-like perivascular lymphocytic infiltration, and solar elastosis were present in the dermis (Fig. 2). The findings were compatible with interface dermatitis and since necrotic keratinocytes were dominant; phototoxic dermatitis was the favored diagnosis. He was started topical mometasone furoate treatment resulting in some improvement.

**Figure 1:**
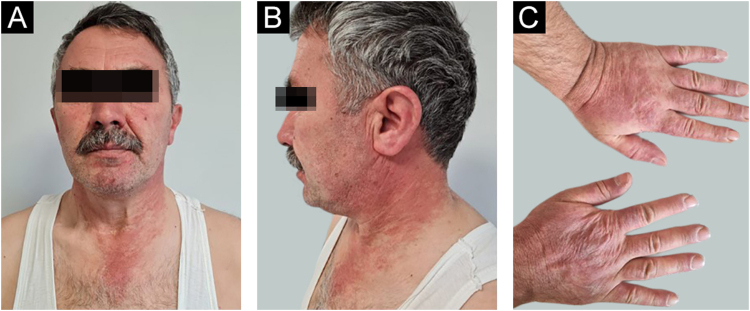
Erythematous, well-defined, photodistributed patches and plaques involving the neck and face (A–B) along with erythematous, violaceous patches upon the hands (C).

**Figure 2:**
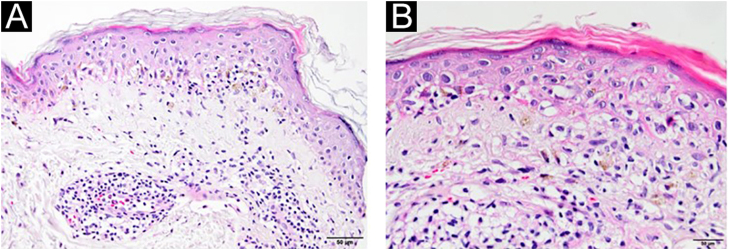
Epidermal basal vacuolar degeneration, coat-sleeve like perivascular inflammation (A) (Hematoxylin & eosin, ×200). Necrotic keratinocytes and lymphocytes in the epidermis (satellite cell necrosis), melanin incontinence and extravasated erythrocytes (B) (Hematoxylin & eosin, ×400).

Vandetanib is an oral chemotherapeutic agent that shows its effect by inhibiting several kinases and thereby interfering with tumor growth and angiogenesis.^1^ Although vandetanib is reported to have systemic side effects such as myalgia, fatigue, hypertension, and transaminitis,^1^ dermatologic manifestations such as pruritus, xerosis and acneiform cutaneous eruption are also associated with vandetanib.^2^ Photosensitive drug eruptions can be classified into phototoxic and photoallergic forms.^3^ Increased oxidative stress and inflammation due to UV exposure and EGFR inhibition are held responsible for the development of photosensitive drug reactions.^4^ Photosensitizer drugs become activated after UV absorption leading to cytotoxic cell injury and increased oxidization which causes phototoxicity.^4^ Incriminated phototoxic agents are furocoumarins, tetracyclines, fluoroquinolones, antifungals, antimalarials, non-steroidal anti-inflammatory drugs, and chemotherapeutic agents.^4^ Yin et al.^2^ reported a case of phototoxic drug eruption on the face and neck developing in a patient under vandetanib treatment for MTC. Other than phototoxicity, vandetanib is also shown to be associated with photoallergic dermatitis, photoonycholysis, cutaneous brown/blue-gray pigmentation, folliculitis, hypertrichosis, periorbital edema, perifollicular dark blue/gray macules.^5^ Topical corticosteroids, sun avoidance, and sunscreen are usually effective for mild to moderate photosensitive reactions. As new targeted therapy agents for various malignancies are developed, plentiful dermatologic side effects will likely be encountered.

## Financial support

None declared.

Informed consent for publication of medical images was taken from the patient. The patient authorized the use of his pictures in the article.

## Authors’ contributions

Ecem Bostan: Conceptualization; visualization; writing the original draft.

Duygu Gulseren: Conceptualization, supervision, and editing

Ozay Gokoz: Conceptualization, supervision, and editing.

## Conflicts of interest

None declared.
